# A Mouse Model of Schnyder Corneal Dystrophy with the N100S Point Mutation

**DOI:** 10.1038/s41598-018-28545-0

**Published:** 2018-07-05

**Authors:** Fei Dong, Xueting Jin, Michelle A. Boettler, Harrison Sciulli, Mones Abu-Asab, Christina Del Greco, Shurong Wang, Yueh-Chiang Hu, Maria M. Campos, Shelley N. Jackson, Ludovic Muller, Amina S. Woods, Christian A. Combs, Jianhua Zhang, Michael L. Nickerson, Howard S. Kruth, Jayne S. Weiss, Winston W. Kao

**Affiliations:** 1 Department of Ophthalmology, University of Cincinnati, Cincinnati, OH USA; 2 Laboratory of Experimental Atherosclerosis, National Heart, Lung, and Blood Institute, NIH, Bethesda, MD USA; 3 Histopathology Facility, National Eye Institute, NIH, Bethesda, MD USA; 4 Ophthalmology, the Second Hospital of Jilin University, Changchun, Jilin, China; 5 Transgenic Animal and Genome Editing Core, Cincinnati Children’s Hospital Medical Center, Cincinnati, OH USA; 6 Structural Biology Core, National Institute of Drug Abuse, NIH, Baltimore, MD USA; 7 Light Microscopy Core Facility, National Heart, Lung, and Blood Institute, NIH, Bethesda, MD USA; 8 Laboratory of Translational Genomics, National Cancer Institute, NIH, Bethesda, MD USA; 9 Department of Ophthalmology, Pathology and Pharmacology, Louisiana State University School of Medicine, Louisiana State University Eye Center, Louisiana State University Health Sciences Center, New Orleans, LA USA

## Abstract

Schnyder corneal dystrophy (SCD) is a rare autosomal dominant disease in humans, characterized by abnormal deposition of cholesterol and phospholipids in cornea caused by mutations in the *UbiA* prenyltransferase domain containing 1 (*UBIAD1*) gene. In this study, we generated a mouse line carrying *Ubiad1* N100S point mutation using the CRISPR/Cas9 technique to investigate the pathogenesis of SCD. *In vivo* confocal microscopy revealed hyper-reflective dot-like deposits in the anterior cornea in heterozygotes and homozygotes. No significant change was found in corneal epithelial barrier function or wound healing. Electron microscopy revealed abnormal mitochondrial morphology in corneal epithelial, stromal, and endothelial cells. Mitochondrial DNA copy number assay showed 1.27 ± 0.07 fold change in homozygotes versus 0.98 ± 0.05 variation in wild type mice (P < 0.05). Lipidomic analysis indicated abnormal metabolism of glycerophosphoglycerols, a lipid class found in mitochondria. Four (34:1, 34:2, 36:2, and 44:8) of the 11 glycerophosphoglycerols species identified by mass spectrometry showed a significant increase in homozygous corneas compared with heterozygous and wild-type mouse corneas. Unexpectedly, we did not find a difference in the corneal cholesterol level between different genotypes by filipin staining or lipidomic analysis. The *Ubiad1*^*N100S*^ mouse provides a promising animal model of SCD revealing that mitochondrial dysfunction is a prominent component of the disease. The different phenotype in human and mouse may due to difference in cholesterol metabolism between species.

## Introduction

Schnyder corneal dystrophy (SCD) is a rare autosomal dominant disease characterized by an abnormal accumulation of cholesterol and phospholipids within cornea, ultimately resulting in decreased visual acuity^1,2^. Corneal signs can manifest as early as the first decade of life. There is a progressive corneal opacification which occurs in a predictable pattern dependent on age^3^. Characteristic subepithelial corneal crystals are found in 50% of affected patients^3,4^.

The gene UbiA Prenyltransferase Domain Containing 1 (*UBIAD1*), also known as *TERE1(transitional epithelial response protein 1*), on human chromosome 1p36 locus is the causative gene of this corneal dystrophy^2,5,6^. Studies show that UBIAD1 is a prenyltransferase participating in menaquione-4 (i.e., vitamin K_2_) synthesis and cholesterol metabolism^2,7^. It is also an antioxidant enzyme regulating eNOS (endothelial nitric oxide synthase 3) activity^8^. To date, twenty five independent putative mutations have been reported p.A97T, p.G98S, p.N102S, p.T103I, p.D112G/N, p.D118G, p.R119G, p.L121F/V, p.V122E/G, p.S171P, p.Y174C, p.T175I, p.G177R/E, p.K181R, p.G186R, p.L188H, p.N232S, p.N233H, p.D236E, p.D240N and p.I245N. Among them, the N102S mutation is a hot spot mutation, accounting for 39% of all cases^9^. However, the pathogenesis of SCD is not clear. Currently, there is no SCD animal model available for researchers and clinicians to study this disease. Here we report an *Ubiad1* mutant mouse line that we created by the CRISPR/Cas9 gene editing technique to carry a missense mutation N100S, corresponding to the human UBIAD1 N102S mutation.

## Results

### Generation of *Ubiad1*^*N100S*^ mice

**Figure 1:**
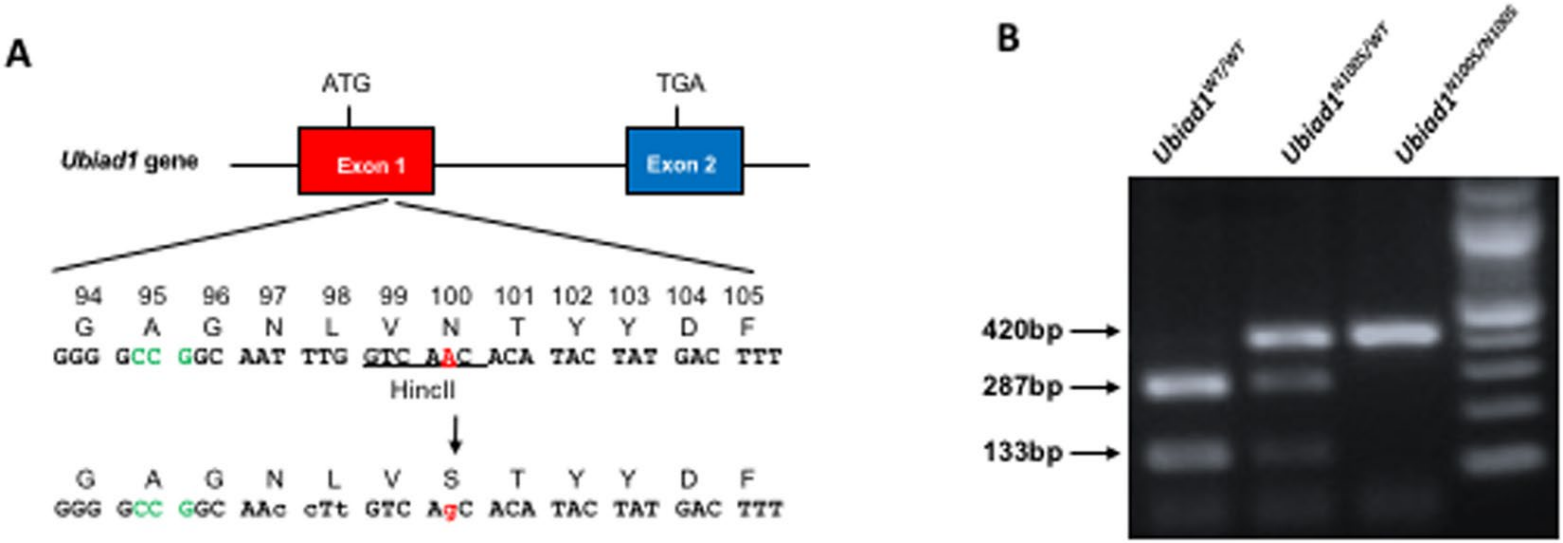
(**A**) Create an N100S mutation using CRISPR-Cas9 system with sgRNA sequence: GTATGTGTTGACCAAATTGC**CGG**. Four nucleotide substitutions are shown in lower case, N100S mutation is highlighted in red. PAM is highlighted as green. (**B**) To identify the genotypes of *Ubiad1*^*N100S*^ mice, a 420 bp PCR product was amplified from purified tail DNA and subjected to HincII digestion. Homozygotes (*Ubiad1*^*N100S/N100S*^), heterozygotes (*Ubiad1*^*N100S/WT*^) and *Ubiad1*^*WT/WT*^ have 1 (size 420 bp), 3 (size 420 bp, 287 bp and 133 bp), and 2 (size 287 bp and 133 bp) bands respectively after HincII digestion. WT, wild-type; Het, heterozygous; Homo, homozygous.

Three sgRNAs were designed for *Ubiad1*^*N100S*^ gene targeting and tested for the cleavage efficiencies in mK4 cells (see the guide RNA sequences and efficiencies in Table 1). sgRNA 3, with the highest cleavage efficiency, together with SpCas9 mRNA and donor oligos, which harbored 5′-76 bp and 3′-60 bp homology arms, were injected to C57BL/6 N mouse zygotes to generate *Ubiad1*^*N100S*^ knock in mice. Schematic of the generation of *Ubiad1*^*N100S*^ mice were shown in Fig. 1A. One of the founder mice was mated with C57BL/6 N wild-type mice and then inbred for heterozygous and homozygous offspring. By digesting the PCR product with wild-type DNA specific restriction enzyme HincII, homozygotes (*Ubiad1*^*N100S/N100S*^), heterozygotes (*Ubiad1*^*N100S/WT*^) and wild-type (*Ubiad1*^*WT/WT*^) could be determined by the number and size of bands, meaning homozygotes have one resistant band (420 bp, the size of PCR product), heterozygotes have three bands including one resistant band and two digested bands (size 420 bp, 287 bp and 133 bp respectively), while wild-type mice have two digested bands (size 287 bp and 133 bp) after HincII digestion (Fig. 1B). Full-length gel of mice tail DNA PCR genotyping before being cropped and rearranged was shown in Supplementary Fig. 1. Both *Ubiad1*^*N100S/WT*^ and *Ubiad1*^*N100S/N100S*^ mice were viable and fertile.

**Table 1:** sgRNA sequence and cleavage efficiency.

Guide RNA	Sequence	Efficiency (%)
sgRNA1	TGTACACGGGGCCGGCAATT**TGG**	8.6
sgRNA2	GTCCTGGCTGTACACGGGGC**CGG**	12.3
sgRNA3	GTATGTGTTGACCAAATTGC**CGG**	12.9
Tet2 (control)	GAAAGTGCCAACAGATATCC**AGG**	13.4

### Minor morphological alterations of the cornea of *Ubiad1*^*N100S*^ mice

#### No signs of crystal or opacity from stereomicroscopy and HE staining

**Figure 2:**
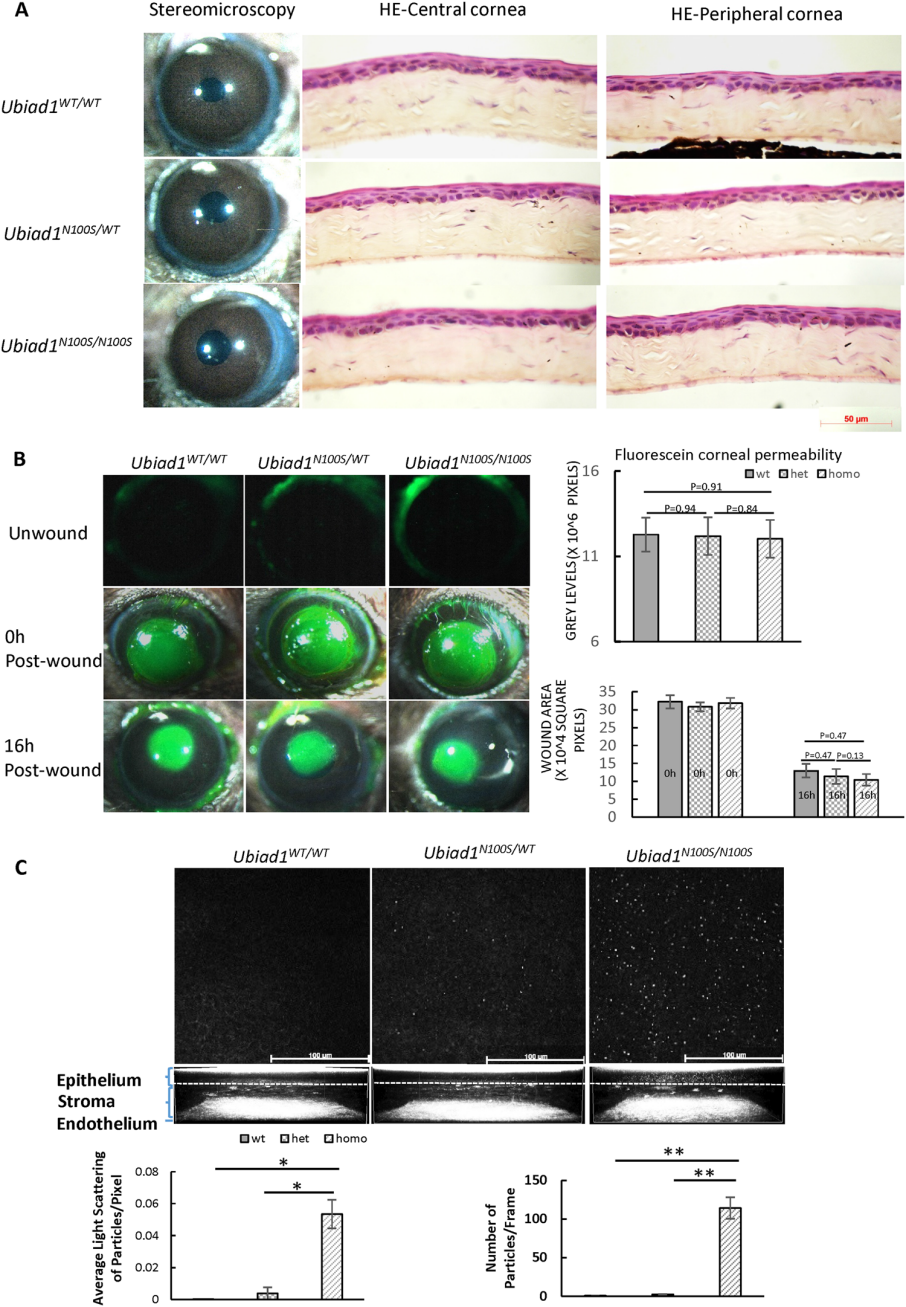
Minor morphological alterations of the cornea of *Ubiad1*^*N100S*^ mice. (**A**) Stereomicroscopy and H&E staining showed intact and clear cornea and normal cornea morphology for the *Ubiad1*^*N100S*^ mutant. Mice were six-month-old of mixed genders. (**B**) No Corneal epithelial barrier function and wound healing change. Representative images of corneal epithelial fluorescein staining of unwound, 0 h and 16 h after 2 mm debridement wounds of *Ubiad1*^*WT/WT*^, *Ubiad1*^*N100S/WT*^ or *Ubiad1*^*N100S/N100S*^ mice. Grey levels of the images of unwound cornea revealed no significant difference in corneal epithelial barrier during different genotypes. The average defect remaining in *Ubiad1*^*N100S/N100S*^, *Ubiad1*^*N100S/WT*^ and *Ubiad1*^*WT/WT*^ mice showed healing of epithelial debridement was similar between three groups. (**C**) *In vivo* confocal microscopy analysis of corneal deposits at 6 weeks. Representative extended focus images combined 5 confocal sections of basal epithelium to show the deposits within the cornea. Quantification of corneal deposit light scattering, and particle number was determined as described under the Methods. The two-tailed Student’s *t*-test was used to analyze the light scattering and particle numbers. Data are presented as mean ± SD. *p < 0.05, **p < 0.01. Mice were of mixed genders.

Unlike human SCD patients, stereomicroscopic examination revealed intact corneas with no crystals or opacities or other abnormalities in *Ubiad1*^*N100S/WT*^ and *Ubiad1*^*N100S/N100S*^ mice (Fig. 2A left panel). HE staining showed normal morphology of central and peripheral cornea from 6-month-old mice of all three groups with normal multilayered epithelium, regular keratocyte distribution in the stroma, and an intact endothelium.

#### No abnormality in corneal epithelial barrier function and wound healing

Little or no fluorescein staining was observed in corneas of *Ubiad1*^*WT/WT*^, *Ubiad1*^*N100S/WT*^ or *Ubiad1*^*N100S/N100S*^ mice (Fig. 2B) showing corneal epithelial barrier function was intact in different genotype mice. To test whether the initial wound healing responses vary among different genotypes, 2 mm manual debridement wounds were performed and the corneas allowed to heal for 16 hours. Healing of epithelial debridement was similar between *Ubiad1*^*WT/WT*^, *Ubiad1*^*N100S/WT*^ and *Ubiad1*^*N100S/N100S*^ mice at 16 hours (Fig. 2B). Though the average defect remaining of *Ubiad1*^*N100S/N100S*^ mice was slightly smaller than that of *Ubiad1*^*WT/WT*^ and *Ubiad1*^*N100S/WT*^ mice, there is no statistically significance. This indicates that *Ubiad1*^*N100S*^ minimally affects epithelial healing in the mouse cornea.

#### Deposits in the cornea of *Ubiad1*^*N100S*^mice

Corneas from five mice of each genotype were subjected to *in vivo* confocal microscopy at the age of 6 weeks (Fig. 2C). Only images of the right eye were used for statistical analysis to exclude the variation caused by position and scanning angle. *In vivo* confocal microscopic images showed highly reflective dot-like deposits scattered in the intermediate and basal corneal epithelium of *Ubiad1*^*N100S/N100S*^ mice as early as 6 weeks of age, while *Ubiad1*^*N100S/WT*^ mice had sporadic deposits, and wild type mice had undetectable deposits at the same age. Corneal stroma of *Ubiad1*^*N100S/N100S*^ and *Ubiad1*^*N100S/WT*^ mice show trace amount of deposits at 6 weeks, and the level of the deposits increased progressively with age in both genotypes (data not shown). Endothelium in all genotypes displayed normal morphology (Representative videos of *in vivo* confocal microscopy from epithelium to endothelium are available in supplementary. The stroma of *Ubiad1*^*WT/WT*^, *Ubiad1*^*N100S/WT*^ and *Ubiad1*^*N100S/N100S*^ were from 00:03.12 to 00:09.35, 00:02.85 to 00:09.97 and 00:03.20 to 00:08.72 respectively. Mice were 6-month-old of mixed genders).

### Mitochondrial damage in *Ubiad1*^*N100S*^ cornea

**Figure 3:**
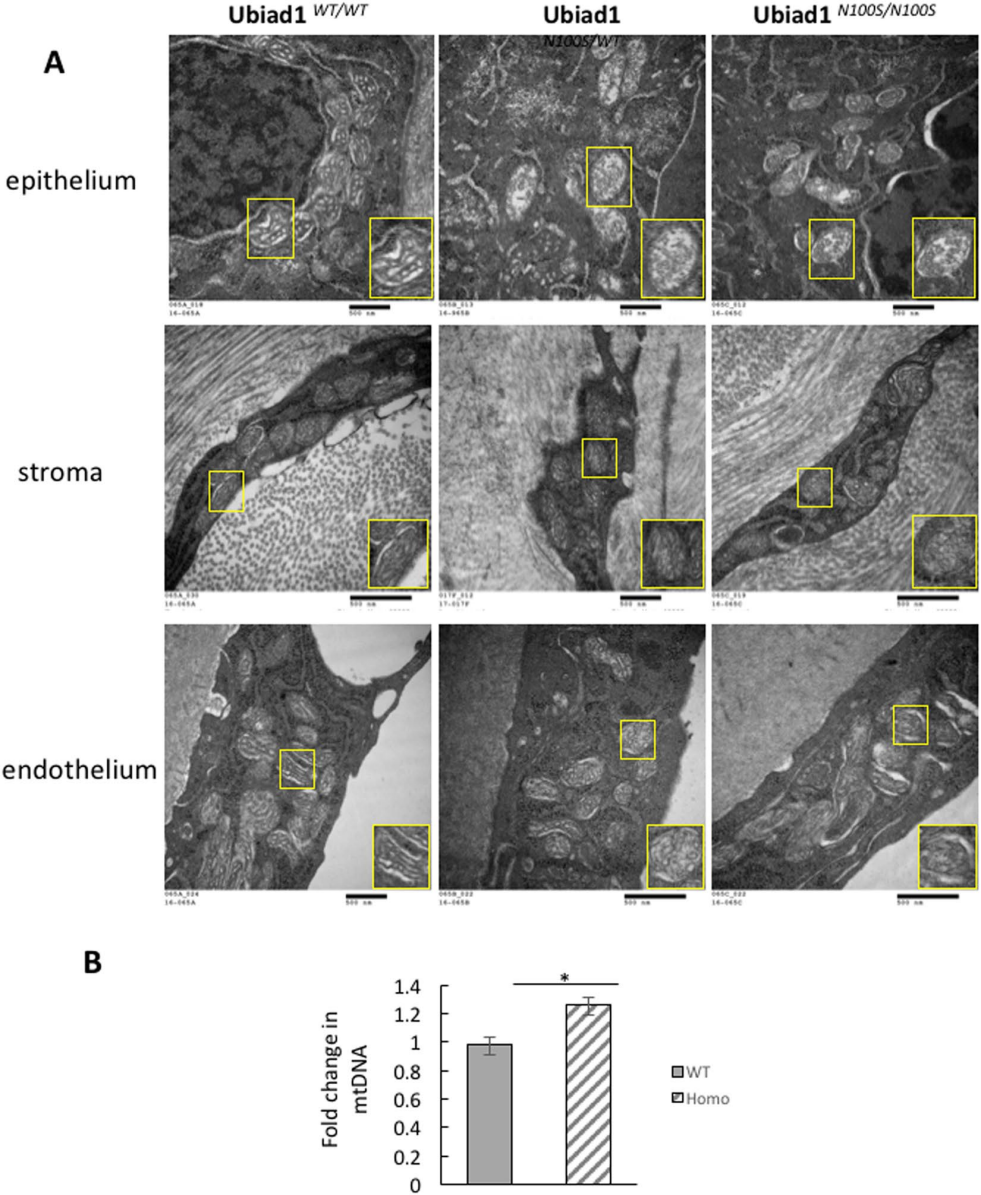
Mitochondria damage in Ubiad1^N100S^ cornea. (**A**) Electron Microscopy showed mitochondrial degeneration in the corneal epithelial cells, stroma keratocytes, and endothelium of heterozygous and homozygous mice. Inset at lower right in each micrograph shows an individual mitochondrion at higher magnification. Mice were 4-month-old males. (**B**) Quantification of mitochondrial DNA copy number change in wild type mice and *Ubiad1*^*N100S/N100S*^ mice by real-time PCR. The fold change was 0.98 in wild type mice versus 1.27 in *Ubiad1*^*N100S/N100S*^ mice, P < 0.05. Mice were of mixed genders.

Electron microscopy images revealed abnormal mitochondrial morphology in the epithelium, stromal keratocytes and the endothelium (Fig. 3A). These changes were most severe in the epithelial mitochondria. The mitochondrial damage was characterized by conversion of the normal distinct mitochondrial cristae in the wild-type mouse corneal cells into less distinct cristae in the mutant mice corneal cells where the cristae appeared blurred. Often, the cristae were spherical due to loss of their normal linear and curvilinear shape.

Mitochondrial DNA copy number of wild type and *Ubiad1*^*N100S/N100S*^ mice was analyzed by comparison of mitochondrial (mt) and nuclear (n) DNA measured by real-time PCR. Compare to the wild type mice, an increase (1.27 ± 0.07 fold change) in mtDNA/nDNA took place in *Ubiad1*^*N100S/N100S*^ cornea while no significant (0.98 ± 0.05 -fold change) was found in wild type mouse cornea (P < 0.05) (Fig. 3B).

There was no evidence by electron microscopy of intracellular or extracellular lipid deposits in wild-type or mutant corneas. Also, filipin staining did not reveal any difference in cholesterol staining between wild-type and mutant mouse corneas (Supplementary Fig. 2). In each case, the epithelium showed intense staining, while the stroma showed very little staining.

Lastly, unstained cornea cryosections were examined by polarization microscopy and there was no birefringence which indicated absence of any cholesterol crystals.

### Lipidomic analysis

In positive ion mode, we assigned 95 lipid species as [M + H]^+^ mass peaks except for cholesterol [M-H20 + H]^+^. The number of species per lipid class were as follows: 47 glycerophosphocholines (PCs), 35 glycerophosphoethanolamines (PEs), 12 sphingomyelins (SMs), and cholesterol (CHL). In negative ion mode, we assigned 11 lipid species [31 PEs, 27 glycerophosphoserines (PSs), 14 glycerophosphoinositol (PI), 12 glycerophosphatidic acid (PA), 11 glycerophosphoglycerol (PG), 8 lysoglycerolphosphoethanolamines (LPEs), 3 ceramides (Cer), 2 lysoglycerophosphoserine (LPS), 2 lysophosphatidic acid (LPA), 1 lysoglycerophosphoinositol (LPI), and 1 hexosylsphingosine (HexSph). All lipid species were detected and assigned as [M-H]^−^ mass peaks.

**Figure 4:**
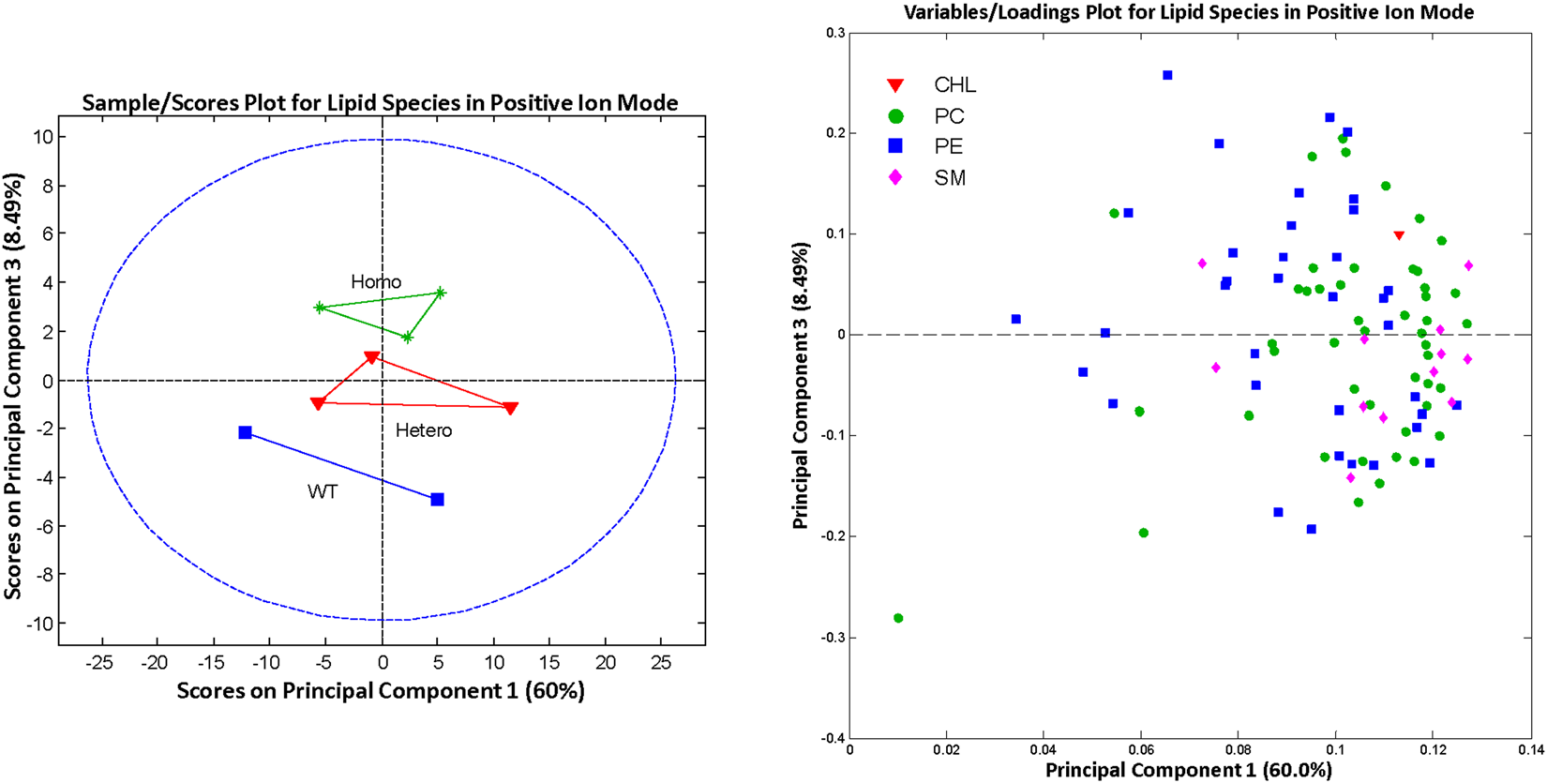
Principal component analysis of lipid species identified by mass spectroscopy in positive ion mode. Outliers from the origin of the loadings plot heavily influence the separation of groups on the scores plot. WT, wild-type; Hetero, heterozygous; Homo, homozygous; CHL, cholesterol; PC, phosphatidylcholine; PE, phosphatidylethanolamine; SM, sphingomyelin.

**Figure 5:**
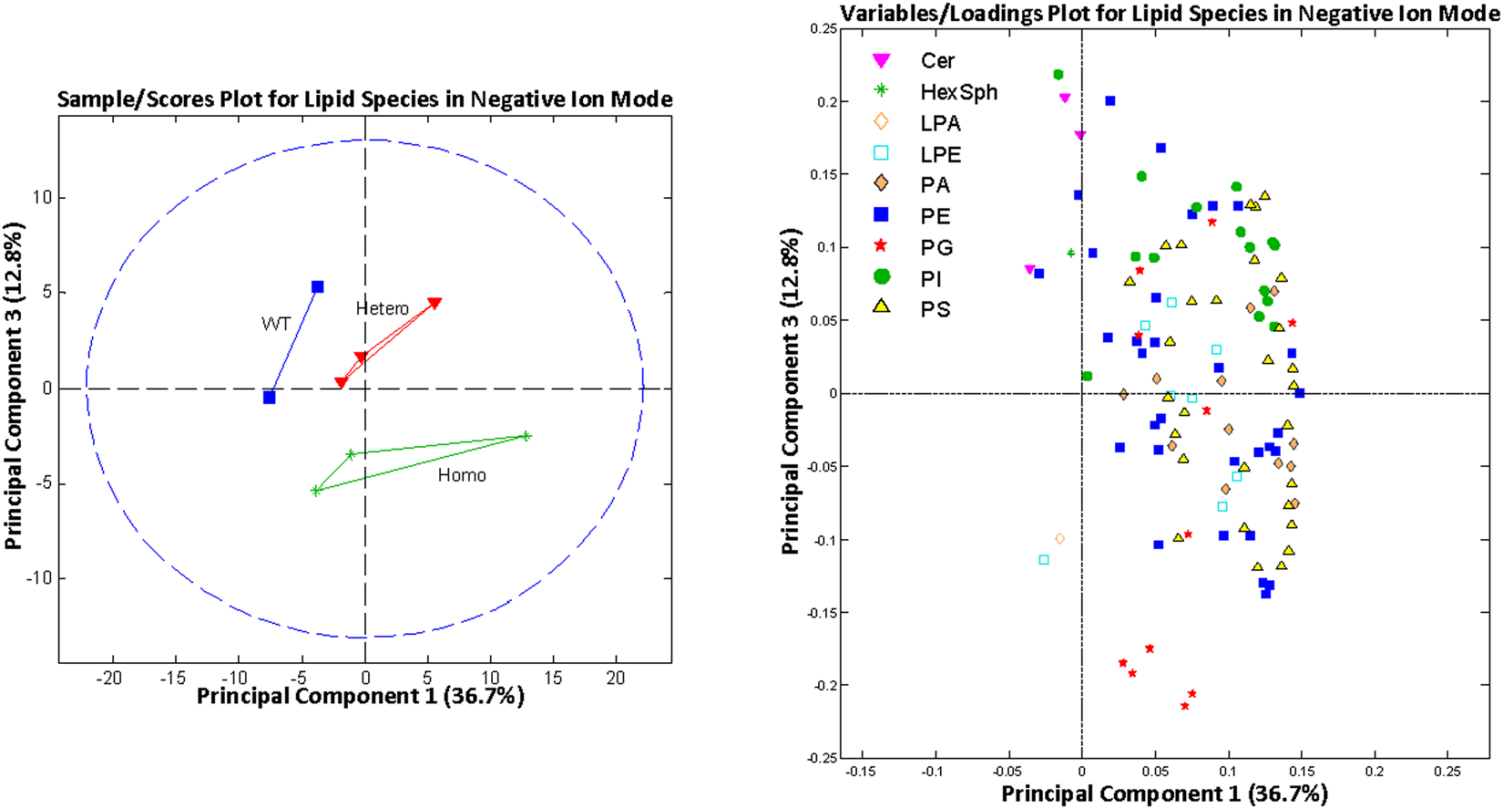
Principal component analysis of lipid species identified by mass spectroscopy in positive ion mode. Outliers from the origin of the loadings plot heavily influence the separation of groups on the scores plot. WT, wild-type; Hetero, heterozygous; Homo, homozygous; CER, ceramide; HexSph, hexosylsphingosine; LPA, lysophosphatidic acid; LPE, lysoglycerolphosphoethanolamine; PA, glycerophosphatidic acids; PE, phosphatidylethanolamine; PG, glycerophosphoglycerol; PI, glycophosphoinositols; PS, glycerophosphoserines.

**Figure 6:**
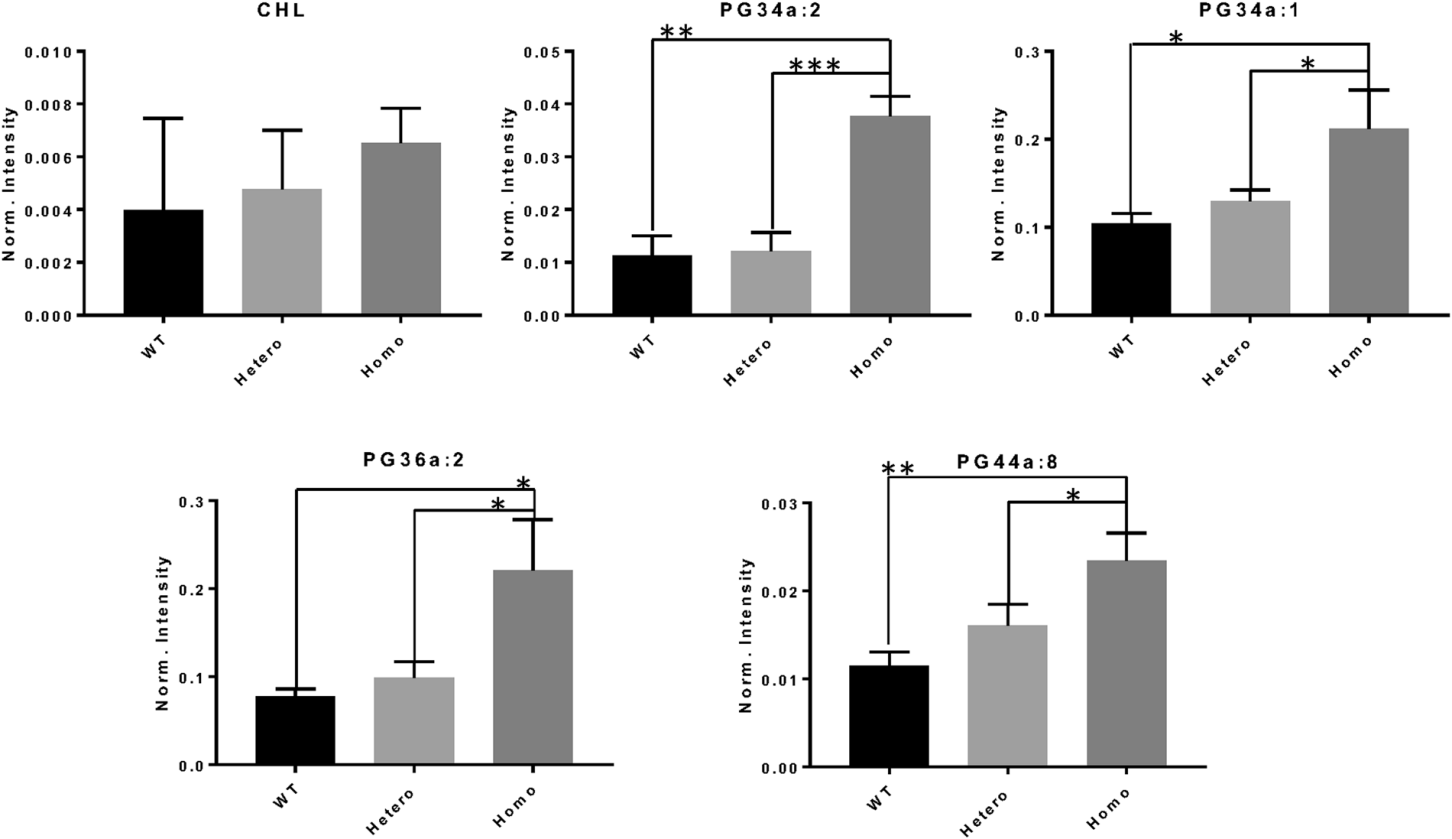
Comparison of the normalized intensity levels for lipid species, mean ± SD, for each of the three mouse genotypes. Statistical differences were determined using one-way ANOVA and Tukey posttests. *p < 0.05, **p < 0.01, ***p < 0.001. Norm, normalized; WT, wild-type; hetero, heterozygous; homo, homozygous; CHL, cholesterol; PG, glycerophosphoglycerol.

The 3 genotypes could be separated by principal component analysis (Figs 4 and 5). Most lipid classes did not show any statistically significant differences among the genotypes. This included cholesterol, the major lipid that is increased in human SCD corneas (Fig. 6). However, for the PG class, 4 (34:1, 34:2, 36:2, and 44:8) out of the 11 identified PG species showed a significant increase in homozygous mutant mouse corneas compared with heterozygous and wild-type mouse corneas (Fig. 6).

## Discussion

Schnyder corneal dystrophy (SCD) is an autosomal dominant disease in humans caused by mutations in the *UBIAD1* gene. It is characterized by abnormal deposition of cholesterol and phospholipids in the cornea resulting in corneal opacification ultimately causing visual loss. Corneal transplantation is often required to restore acceptable vision. Approximately 50% of patients also demonstrate subepithelial crystals which are assumed to be comprised of cholesterol^3^. Although the exact pathogenesis of SCD is not understood, SCD is assumed to be a local metabolic abnormality of the cornea. This is supported by a study of 13 affected patients in which there was relentless progression of the corneal opacification during a 9 year follow up, even when systemic cholesterol had been normalized^10^.

By mimicking the hot spot SCD mutation N102S in humans, we generated an *Ubiad1*^*N100S*^ mouse line, the first animal model for SCD. Dot-like deposits in the anterior cornea of *Ubiad1*^*N100S/WT*^ and *Ubiad1*^*N100S/N100S*^ mice were demonstrated by HRTII scanning as early as 6 weeks. The accumulation of these deposits was progressive, which is consistent with progression of deposits in human patients.

The source of cholesterol that accumulates in SCD corneas is not known. An early study of corneal accumulation of radiolabeled cholesterol injected into an SCD patient showed that the radiolabeled cholesterol accumulated within the cornea at a higher specific activity than that within the plasma (the cornea had been removed during corneal transplant surgery)^11^. This suggests that circulating plasma lipoproteins are the source of the cholesterol.

Alternatively, UBIAD1 is a prenyltransferase that has the potential to regulate cholesterol synthesis by facilitating sterol-dependent degradation of the rate limiting cholesterol biosynthetic enzyme, HMG CoA reductase, to which it binds^7,12^. The SCD mutated N102S UBIAD1 blocks sterol-dependent degradation of HMG CoA reductase, suggesting a different explanation for cholesterol buildup in which unregulated cholesterol synthesis could be the source of accumulated corneal cholesterol.

Expression of ectopic UBIAD1 decreases the cholesterol content of several tested cell lines^13^, but whether this decrease is due to a decrease in cholesterol synthesis, an increase in cholesterol uptake, or an increase in cholesterol efflux from cells was not determined. On the other hand, knockdown of *UBIAD1* in HepG2 cells shows no effect on the cholesterol content of these cells^14^. Also, expression of SCD mutated N102S UBIAD1 in human umbilical artery endothelial cells does not alter cellular cholesterol levels compared to cells with normal *UBIAD1* expression^8^. This suggests that if mutated UBIAD1 does lead to increased synthesis of cholesterol, then this cholesterol must be effluxed from the cell.

Although we observed dot-like deposits in mutant mice by HRTII laser scanning, we could not find any evidence that cholesterol accumulated within the mutant corneas. Histochemical staining of cholesterol with filipin and cholesterol lipid levels detected by mass spectrometry did not show any evidence of cholesterol difference between wild-type and mutant mice. Also, the dot-like deposits did not give rise to birefringence, indicating these structures were not the cholesterol crystals, which occur in about 50% of human SCD corneas^3,4^. Furthermore, no evidence of lipid deposits was detected in mutant corneas by electron microscopy, although extracellular liposomal-like lipid deposits are evident in SCD using electron microscopy^1,11,15–17^. It remains possible that lipid deposition would occur if the mice were of sufficient age, 4 months-old mice might be too young to develop corneal crystals and show cholesterol difference. In future studies, phenotypes of older mice (≥1 year-old) and special fat diets fed mice will be included to further characterize mutants. However, cholesterol metabolism is different between human and mouse. First, mice, particularly the C57BL/6 mouse strain used in our study, have lower cholesterol levels than humans^18^. Moreover, the major lipoprotein in mice are high-density lipoproteins (HDL) which are known as “good cholesterol”, have high protein/lipid ratios and facilitate reverse cholesterol transport back to the liver. In contrast, the predominant lipoproteins in humans are low-density lipoproteins (LDL), which are considered as “bad cholesterol” and contribute to atherosclerotic plaque formation. Correspondingly, mice have very low levels of apolipoprotein B (ApoB), which is the primary apolipoprotein of LDL^19^. Furthermore, cholesteryl ester transfer protein (CETP) which facilitates the transfer of cholesterol esters from HDL to LDL in humans is not present in mice^20^. Consequently, metabolic differences in cholesterol metabolism between the mice and human could potentially contribute to distinct corneal phenotypes between the two species. In any case, the identity of the dot-like deposits in SCD mutant mice detected by HRTII scanning remains to be determined.

UBIAD1 is known to function as an antioxidant and has been localized to different cellular compartments in different cells. In human umbilical endothelial cells, UBIAD1 is localized within the Golgi apparatus where it likely functions to synthesize its prenylation product, CoQ10. CoQ10 can protect against lipid and protein oxidation^8^. Thus, although increased levels of products of reactive nitrogen and oxygen species, nitrotyrosine, and malondialdehyde, respectively, were found in SCD patients^21^, it is unlikely that this results from impaired Golgi UBIAD1-mediated synthesis of CoQ10. This is because mutated N102S *UBIAD1* expressed in zebrafish and human endothelial cells does not synthesize less CoQ10 compared with that of normal UBIAD1^8^.

A major finding in the *Ubiad1* mutant mouse that we report is the effect of the N100S mutation on mitochondrial morphology and biosynthesis. Mitochondria showed evidence of dysfunction as evidenced by disorganization and less distinct cristae inner membranes, similar to what has been described previously for HepG2 cell lines in which *UBIAD1* was genetically silenced^14^. There is also one report of mitochondrial abnormality observed by electron microscopy in a patient with SCD^22^. The finding of elevated levels of the PG class of phospholipids in the homozygous mutants is consistent with mitochondria dysfunction because PG lipids are found exclusively in the mitochondria where they are synthesized^23^.

The mtDNA is a double-stranded circular molecule located in mitochondrion, encoding 37 genes essential for normal mitochondrial functioning. Its biosynthesis is independent of the nuclear DNA replication. mtDNA copy number, measured by the ratio of mitochondrial (mt) and nuclear (n) DNA, is an indirect marker of mitochondrial function and may change under different physiological or environmental conditions^24^. Mitochondrial dysfunctional has been hypothesized to contribute to oncogenesis. mtDNA copy number has been found changed in almost all human cancers, however increased in some cancers such as acute lymphoblastic leukemia, colorectal carcinoma, but reduced in most tumor tissues of advanced gastric cancer, breast cancer *et al*.^25^. In aged human tissues, mtDNA content were found either elevated or depleted or remained more or less the same^26–29^. Under stimulation or pathogenesis such as gamma-irradiation, high glucose-induce or diabetes, mtDNA copy number were found increased^30–32^, might be due to an acute elevation of oxidative stress. In *Ubiad1*^*N100S/N100S*^ mice, the elevated mtDNA copy number may reflect a cellular response to compensate for damaged mitochondria.

Morphologic abnormality of mitochondria was present in both heterozygous and homozygous mutant mice, while PG elevation was present in only the homozygous mice. This suggests that elevated PG levels did not cause the abnormal mitochondrial morphology, but rather that both were the result of some other aspect of mitochondrial metabolism that affected mitochondria structure with greater sensitivity than it affected mitochondrial PG metabolism. All published human patients with SCD are heterozygous for the mutation, which points toward the significance of the abnormal mitochondrial morphology also observed in heterozygous mice.

Besides synthesizing CoQ10, UBIAD1 synthesizes vitamin K_2_ in many tissues^33^. Initially this activity of UBIAD1 was localized to the endoplasmic reticulum^33^. Previously we demonstrated that UBIAD1 was localized within mitochondria of cultured human cornea keratocytes^9^. Subsequently, it was shown that UBIAD1 localizes to mitochondria in a human bladder cancer cell line^34^, and its homolog is present within *Drosophila* mitochondria where it also functions to synthesize vitamin K_2_ (i.e., menaquinone-4)^33,35^. Vitamin K_2_ in turn functions as an electron carrier contributing to mitochondrial production of ATP and helping to maintain mitochondrial membrane potential^10,13,34,35^. In this regard, we have shown that SCD mutations, including N102S, show impaired synthesis of vitamin K_2_^7^. Thus, vitamin K_2_ deficiency could compromise mitochondrial electron transport in cells of the cornea. This may be of particular importance in the cornea given the exposure of these cells to light, which is known to impair mitochondria electron transport^36^.

## Conclusion

We created a mouse model of SCD revealing that mitochondrial dysfunction is a prominent component of the disease, consistent with the mitochondrial localization of UBIAD1 in cultured corneal keratocytes that we previously reported^9,10^. It remains to be determined whether defective mitochondria function is linked to the abnormal cholesterol deposition that occurs in SCD patients. Mitochondria function is known to be linked to cholesterol metabolism through mitochondria synthesis of cholesterol oxidation products that regulate transcription factors such as LXR, affecting genes that regulate cholesterol trafficking and efflux from cells^7,10,22,37–40^. The effect of UBIAD1 could be mediated also by its known binding to ApoE (functions in cholesterol efflux), HMG-CoA reductase (regulates cholesterol synthesis), sterol O-acyltransferase (esterifies cholesterol in cells), and binding to cholesterol itself ^7,37,38^. Future studies of *Ubiad1* mutant mice will elucidate new links between mitochondrial function and cholesterol metabolism.

## Methods

### sgRNA and DNA donor template design for *Ubiad1*^*N100S*^ gene editing

sgRNAs were designed and preliminarily evaluated according to the previous study^41^. Briefly, three sgRNAs were designed using CRISPR tool (http://crispr.mit.edu) and cloned into a modified pX458 plasmid (Addene plasmid #48138) bearing both sgRNA scaffold backbone (BB) and SpCas9. Their activities were compared side-by-side to Tet2 sgRNA that is known efficiently^42^. Constructed CRISPR-Cas9 plasmids were then transfected into mouse mK4 cells to test individual sgRNA’s editing activity by T7E1 assay^43^. sgRNA sequences and editing efficacy are listed in Table 1. sgRNA3, which had the highest efficacy, was selected for the generation of the *Ubiad1*^*N100S*^ mouse. A DNA template with 76 bp left and 60 bp right homology arms flanking the mutant N100S codon of the Ubiad1 gene had four nucleotide substitutions (Exon1 nt592 [T-to-C, a synonymous mutation], nt593 [T-to-C, a synonymous mutation], nt595 [G-to-T, a synonymous mutation] and nt600 [A-to-G, a missense mutation. The HincII restriction enzyme site in wild-type DNA was destroyed after recombination (Fig. 1A). The *Uibad1* donor sequence was as follows (substitutions are in lower case, N100S mutation is in bold text): 5′CACCAAAGTTCTGTCATCACTCTTTTTGTGGTCAATGCCCTTGGAAAAGTCATAGTATGT**GcT**GACaAggTTGCCGGCCCCGTGTACAGCCAGGACAGCCACTGCACAACCCAACAACAGCCTGGGATCCAGGACACCC3′

### Construction and generation of *Ubiad1*^*N100S*^ mice using Crispr/Cas9

All animal procedures were performed in accordance with the Association for Research in Vision and Ophthalmology (ARVO) Statement for the Use of Animals in Ophthalmic and Vision Research and approved by the Institutional Animal Care and Use Committee(IACUC) of the University of Cincinnati and the National, Heart, Lung, and Blood Institute, NIH (Bethesda, MD). *Ubiad1*^*N100S*^ knock-in mice were generated by homologous recombination through cytoplasmic injection of 100 ng/µl SpCas9 mRNA, 50 ng/µl sgRNA, and 100 ng/µl donor oligonucleotides to C57BL/6 mouse zygotes. Injected zygotes were transferred to pseudopregnant CD1 females immediately. Three independent *Ubiad1*^*N100S*^ knock-in heterozygous founders (F0) were obtained and confirmed by DNA sequencing to carry the designated substitution mutations. One founder was used in the present study.

### Breeding and identification of *Ubiad1*^*N100S*^ mice

The founder mouse was mated with C57BL/6 wild-type (WT) mice (Jackson Laboratory, Bar Harbor, ME) and then interbred for heterozygous and homozygous offspring.

To identify the genotypes of *Ubiad1*^*N100S*^ mice, a 420 bp PCR product was amplified from purified tail DNA (forward: 5′-CTTCAGTGCCTCACTCACC-3′; reverse: 5′- AGGTAGCAGTTTCCAGTTTAGG-3′). PCR products were subjected to HincII (GT(T/C)^▼^(A/G)AC) digestion for 2 hours at 37 °C. Homozygotes (*Ubiad1*^*N100S/N100S*^), heterozygotes (*Ubiad1*^*N100S/WT*^) and wild-type (*Ubiad1*^*WT/WT*^) mice had 1 (size 420 bp), 3 (size 420 bp, 287 bp and 133 bp), and 2 (size 287 bp and 133 bp) bands, respectively, after digestion (Fig. 1B).

### *In vivo* confocal microscopy of cornea

*In* vivo examination of cornea light scatter was performed with the Heidelberg Retinal Tomograph-HRTII Rostock Cornea Module (HRT-II, Heidelberg Engineering Inc., Heidelberg, Germany) according to the manufacturer’s instruction as described previously^44^. Briefly, a drop of GenTeal® Gel (Novartis Pharmaceuticals Corp., East Hanover, New Jersey) was applied to the tip of the HRT-II objective as immersion fluid. After anesthesia, a series of images were collected to cover the whole cornea as a continuous z-axis scan through the entire cornea at 2 µm increments starting from superficial corneal epithelium and ending at the corneal endothelium.

### Quantification of the corneal particle light scattering and number of corneal particles

Average light scattering of wild-type, heterozygotes and homozygotes was calculated using *in vivo* confocal images at the age of 6 weeks. The last five frames of basal epithelial *in vivo* confocal images were selected for quantitative analysis. Light scattering of the particles and numbers of particles were measured using Image J. Briefly, threshold light scattering of the particles, which was calculated by first averaging the maximum brightness values of the controls using Stacks plugin. The average maximum brightness of the controls was set as the minimum threshold, and the maximum brightness was set as 255 (highest value). Light scattering of the particles in the mutant mice was then determined using these parameters. Similarly, particle numbers were analyzed using the same brightness threshold. The two-tailed Student’s t-test (Excel, Microsoft, Redmond, WA) was used to analyze the light scattering and particle numbers. All quantification data are presented as mean ± SD.

### Evaluation of corneal epithelial barrier function

Three months old *Ubiad1*^*WT/WT*^, *Ubiad1*^*N100S/WT*^ and *Ubiad1*^*N100S/N100S*^ mice (n = 4) were anesthetized, and administrated 10 μL of 0.5% fluorescein sodium to conjunctival sac for 1 minute; excess fluorescein was flushed out with PBS. Fluorescein pickup by the cornea was then recorded by stereomicroscope (Carl Zeiss Microscopy GmbH, LA, USA). Grey levels of the images were calculated by ImageJ to evaluate corneal epithelial barrier function.

### Corneal wound healing

Three months old *Ubiad1*^*N100S/N100S*^, *Ubiad1*^*N100S/WT*^ and *Ubiad1*^*WT/WT*^ mice (n = 4 each) were anesthetized. A 2-mm central corneal area was demarcated with a trephine and corneal epithelium was removed manually by Algerbrush® (Ambler Surgical, PA). The cornea was photographed with fluorescein staining at 0 h and 16 h post-surgery. Wound area was calculated by ImageJ.

### Histological analysis

#### HE staining

Cornea specimens were fixed overnight in 4% paraformaldehyde in phosphate-buffered saline (PBS) at 4 °C, followed by paraffin embedding and sectioning. De-paraffinized sections (5 µm) were stained with hematoxylin/eosin (H&E) and examined with a Nikon ECLIPSE E800 microscope.

#### Filipin staining

Filipin staining was performed to detect any cholesterol deposition in the cornea. Mice from three genotypes were euthanized at the age of four months. Corneas were immediately dissected on ice and embedded in Optimal cutting temperature compound (Sakura Finetek, Torrance, CA) without fixation. Cryosections (10 µm thick) were prepared. Sections were stained at room temperature for 75 minutes with freshly made 0.05 mg/mL filipin III solution (Cat F4767, Sigma, St. Louis, MO). Following staining, sections were mounted in ProLong™ Diamond Antifade mounting medium (Thermo Fisher Scientific, Waltham, MA). Confocal microscopic images of filipin fluorescence were obtained with a Zeiss 780 microscope and Plan-Apochromat 63 ×/1.40 oil objective using 355 nm wavelength for excitation and 371 to 501 nm wavelengths for fluorescence emission. The z-stack obtained contained 3 image slices with a total depth of 1.044 µm.

### Electron microscopy

Mouse eyes were quickly removed after euthanasia and immersed into the electron microscopy fixative solution (2.5% glutaraldehyde and 1% paraformaldehyde in phosphate-buffered saline, pH 7.4) for 6 hours at room temperature. Then, the eyes in fixative solution were kept at 4 °C overnight before corneas were cut out from the eye with a 2-mm punch. The corneas were then stored in fixative solution at 4 °C until further processing.

Processing for electron microscopy was carried out as described previously^45^. Briefly, cornea tissue was further fixed in 2% osmium tetroxide, dehydrated with ethanol and propylene oxide, and embedded in epoxy resin. Ultrathin sections (~90 nm thick) were mounted on 200-mesh uncoated copper grids, doubly stained with uranyl acetate and lead citrate, and examined and photographed with a JEOL JEM-1010 transmission electron microscope (JEOL, Peabody, MA).

### Mouse Mitochondrial DNA quantification

Mitochondrial DNA quantification kit was purchased from (Detroit R&D, Detroit, MI), and carried out according to manufacturer’s protocol. Briefly, total DNA (gDNA) was extracted using phenol-chloroform-isoamyl alcohol (25:24:1) as described previously^46^. Corneas from wild type and *Ubiad1*^*N100S/N100S*^ (n = 4, 6 month old mixed genders) were dissected, and digested in 300 µL DNA lysis buffer (10 mM Tris–HCl (pH 8.0), 1 mM EDTA, 0.1% SDS, and Proteinase K) at 55 °C overnight. 300 µl phenol/chloroform/isoamyl alcohol (25:4:1) was added to lysate solutions. The samples were mixed well by vortex and centrifuged (12,000 *g* for 10 min). The supernatant was transferred to a new tube and mixed with 15 µl NaAc (3 M) and 250 µl isopropanol to precipitate DNA, and then centrifuged (12000 *g* for 10 min) to pellet the DNA. After discarding the supernatant, the DNA pellet were washed with 1 ml 70% ethanol, air dried, and dissolved in 25 µL H2O. Mitochondria DNA copy number was quantified by real time PCR with the Mouse Mitochondrial DNA Copy Number Assay Kit according to the manufacturer’s protocol. The real time PCR was performed on the CFX96™ Real-Time PCR Detection System (Bio Rad, Hercules, California). PCR amplification profile was as follows: 95 °C for 10 min, 40 cycles of denaturation at 95 °C for 15 s, annealing at 60 °C for 1 min. The fluorescence threshold CT value was calculated by the Bio-Rad CFX manager. All assays were carried out in triplicate. mtDNA copy number was calculated by the equation 2^−ΔΔCt^.

### Lipidomic analysis

Eyes were immediately removed after euthanasia from 4-month-old female mice. Next, corneas were trephined from the eye with a 2-mm punch and stored at −80 °C. For lipidomic analysis^47^, corneas were brought up to room temperature under vacuum in a desiccator for 30 minutes. Then, corneas were weighed (Table 2) to determine the amount of internal standard to add. Wild-type #68 was a clear outlier as it contained much lower amounts of lipids than any other sample tested, most likely because something else contributed to the weight that did not contain lipids. Thus, data for this cornea was not included in the lipid analysis results.

**Table 2:** Weights of corneas used for lipid analysis.

Sample	Weight (µg)
Wild type #68	506
Wild type #86	108
Wild type #88	146
Heterozygous #84	116
Heterozygous #85	106
Heterozygous #87	104
Homozygous #72	110
Homozygous #75	116
Homozygous #81	112

The internal standard mix consisted of ceramide C10:0/d18:1 (0.4 µg/mL), phosphatidlycholine 20:0 (10 µg/mL), phosphatidylglycerol 20:0 (6 µg/mL) in chloroform/methanol (2:1, v/v), which was added to the sample at 0.5 µL/µg of cornea. Chloroform/methanol (2:1, v/v) was added to the cornea at 1 µL per 1 µg of cornea including the internal standards. The samples were then homogenized, sonicated for 15 minutes, and vortexed for 1 hour. Next, ddH_2_O was added at 1 µL per 4 µg of cornea and vortexed for one minute. The samples were centrifuged for 10 minutes at 1116 x*g*. The lower phase (organic phase, lipid fraction) was removed and evaporated under dry nitrogen. The samples were then resuspended in 5 µL of chloroform and 100 µL of methanol and stored at −20 °C until mass spectrometry analysis.

For mass spectrometry analysis, samples first were diluted 1:1 in 10 mM ammonium acetate in methanol and analyzed on an Orbitrap Velos mass spectrometer (Thermo Fisher Scientific, Waltham, MA) in both positive and negative ion mode with a mass resolution setting of 100,000. In positive ion mode, a m/z range of 360–1100 was used, while in negative ion mode a m/z range of 400–1000 was used. Peak assignments were based upon exact mass with a mass error under 5 ppm. Data were annotated according to our lipid database. Intensities for all annotated lipids in each sample were automatically reported in Excel using R software (version 2.15.1). Data were normalized with the intensity of the internal standards. Data matrices were processed by multivariate analysis using the PLS toolbox (Eigenvector Research Inc., version 6.7, Wenatchee, WA). Univariate analysis was carried out using one-way ANOVA and Tukey posttests with a p-value < 0.05 considered significant^48,49^.

## Electronic supplementary material

in vivo confocal of WT cornea

in vivo confocal of Ubiad1 het cornea

in vivo confocal of Ubiad1 homo cornea

suplementary data
