# Supplementary material for: A Mouse Model of Schnyder Corneal Dystrophy with the N100S Point Mutation
Source: Sci Rep. 2018 Jul 5;8:10219. doi: 10.1038/s41598-018-28545-0 (PMC6033878; doi:10.1038/s41598-018-28545-0)
Supplement: Supplementary file 4 — suplementary data [file 41598_2018_28545_MOESM4_ESM.pdf]

A Mouse Model of Schnyder Corneal Dystrophy with the N100S Point Mutation

Fei Dong[1#], Xueting Jin[2#], Michelle A. Boettler[1], Harrison Sciulli[1], Mones Abu-Asab[3], Christina Del Greco[1], Shurong Wang[1,4], Yueh-Chiang Hu[5], Maria M. Campos[3], Shelley N. Jackson[6], Ludovic Muller[6], Amina S. Woods[6], Christian A. Combs[7], Jianhua Zhang[1], Michael L. Nickerson[8], Howard S. Kruth[2*], Jayne S. Weiss[9*], Winston W. Kao[1*]

1. Department of Ophthalmology, University of Cincinnati, Cincinnati, OH;

2. Laboratory of Experimental Atherosclerosis, National Heart, Lung, and Blood Institute, NIH, Bethesda, MD;

3. Histopathology Facility, National Eye Institute, NIH, Bethesda, MD

4. Ophthalmology, the Second Hospital of Jilin University, Changchun, Jilin, China;

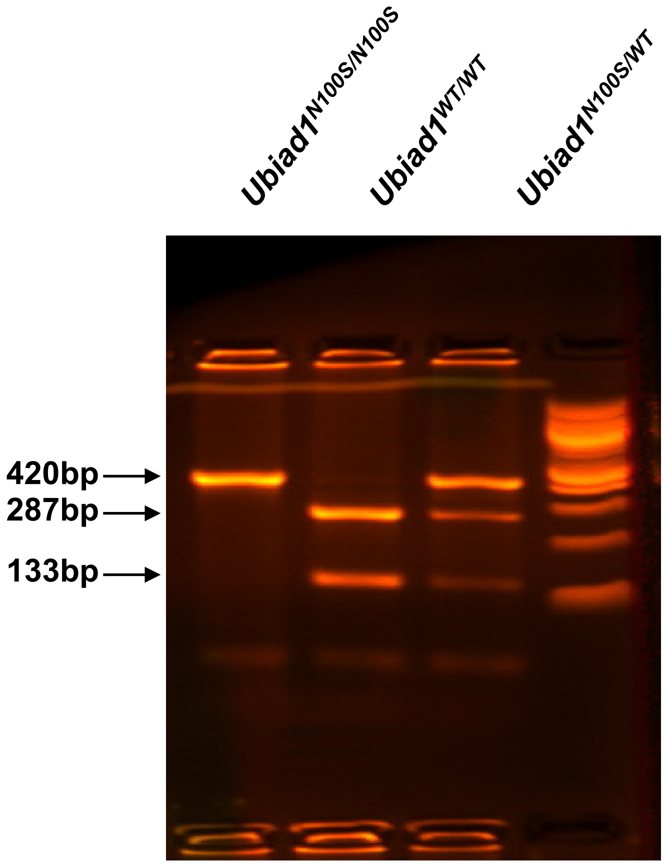

Supplementary Figure 1. Full-length gel of mice tail PCR genotyping before being cropped and rearranged.

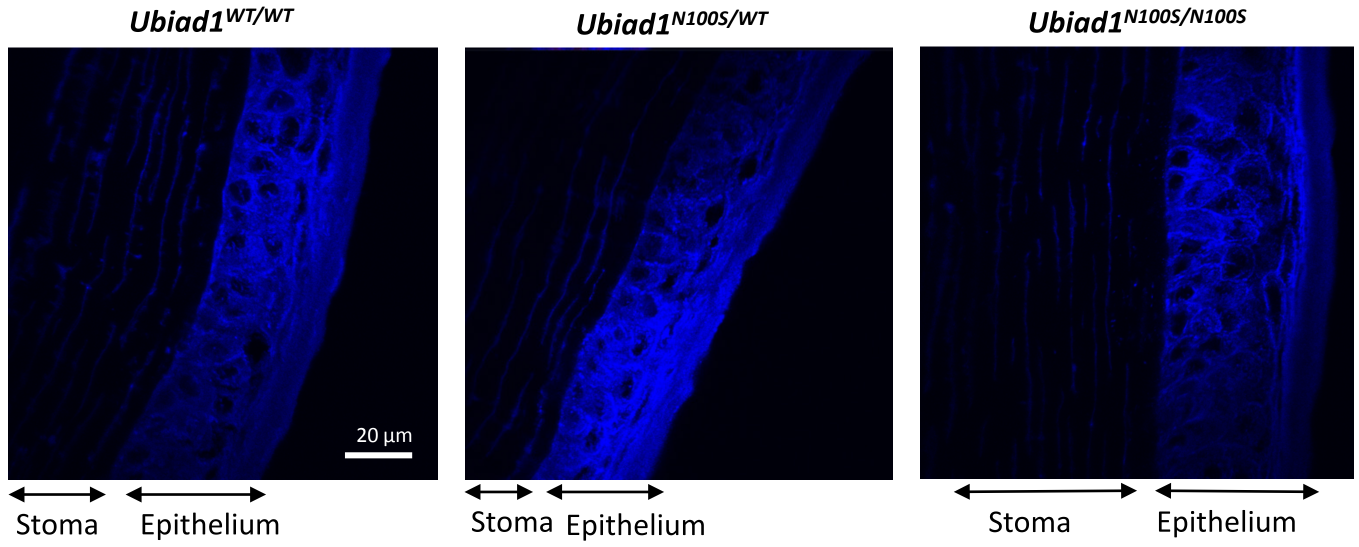

Supplementary Figure 2. Detection of cholesterol with filipin staining. Filipin staining only occurred in the epithelial layer of all mice regardless of genotype, but did not reveal any difference between *Ubiad1*$^{N100S/N100S}$, *Ubiad1*$^{N100S/WT}$*,* and *Ubiad1*$^{WT/WT}$ mice. Mice were 4-month-old males.
